# Dual Autoimmunity: A Case Report of the Sequential Development of Systemic Lupus Erythematosus in a Patient With Anti-MDA5 Dermatomyositis

**DOI:** 10.7759/cureus.83686

**Published:** 2025-05-07

**Authors:** Lorena A López, Luis M Vilá

**Affiliations:** 1 Division of Rheumatology, University of Puerto Rico, Medical Sciences Campus, San Juan, PRI

**Keywords:** anti-mda5 dermatomyositis, dermatomyositis, idiopathic inflammatory myopathies, rituximab therapy, systemic lupus erythematosus, systemic tacrolimus

## Abstract

Dermatomyositis (DM) and systemic lupus erythematosus (SLE) are chronic rheumatic diseases that can affect multiple organ systems. Both conditions share several similarities, including pathogenic mechanisms, clinical manifestations, and pharmacological treatments. However, the coexistence of DM and SLE is rarely encountered in clinical practice. Here, we present the case of a 42-year-old woman who developed DM, characterized by proximal muscle weakness in the upper and lower extremities, dysphagia, heliotrope rash, periungual erythema, erythematous skin lesions on the neck and arms, elevated serum aldolase and creatine phosphokinase (CPK) levels, and positive anti-melanoma differentiation-associated gene 5 (MDA5) antibodies. A skin biopsy confirmed the diagnosis of dermatomyositis. She was initially treated with high-dose corticosteroids and mycophenolic acid, resulting in early improvement. However, three months after the onset of DM, she presented with persistent DM manifestations and the development of new-onset pancytopenia, arthritis, discoid lesions, positive antinuclear antibodies, and C3 hypocomplementemia, consistent with SLE. Rituximab and tacrolimus were added to her regimen of glucocorticoids and mycophenolic acid, and she responded well to therapy, with resolution of all clinical manifestations by four months after starting rituximab and tacrolimus. At the 18-month follow-up, she remained in complete clinical remission from both DM and SLE. This case underscores the complexity of autoimmune diseases. Although the coexistence of DM and SLE is uncommon, healthcare providers should maintain a high index of suspicion in patients presenting with atypical symptoms or overlapping features. It also emphasizes the challenge of managing multiple autoimmune conditions concurrently.

## Introduction

Dermatomyositis (DM) is an idiopathic inflammatory myopathy (IIM) characterized by proximal muscle weakness and distinctive cutaneous manifestations, including heliotrope rash and Gottron’s papules or rash [1]. The estimated prevalence of DM is approximately 13 cases per 100,000 individuals, with a higher frequency observed in women and adults between 40 and 60 years of age [2].

DM is associated with several significant comorbidities that can influence management and prognosis. Among the most notable are malignancies and autoimmune diseases, which may precede, coincide with, or follow the diagnosis of DM [3]. Although SLE has been reported in association with DM, the sequential development of SLE in a patient previously diagnosed with DM is rare and remains poorly characterized in the literature. DM and SLE share overlapping clinical features that can present diagnostic and therapeutic challenges, especially when new symptoms arise, raising concern for either a flare of the primary disease or the emergence of a distinct autoimmune process. Common overlapping features include constitutional symptoms, photosensitivity, erythematous rashes, alopecia, arthralgias, myalgias, myositis, myocarditis, interstitial lung disease (ILD), elevated acute phase reactants, and positive antinuclear antibodies.

Herein, we describe a case of a patient with anti-MDA5 DM who subsequently developed new clinical and serologic findings consistent with SLE, underscoring the importance of vigilant monitoring for evolving autoimmune manifestations and the need for individualized management strategies in patients with overlapping connective tissue diseases.

This case was previously presented as a meeting abstract at the 44th Annual Research and Education Forum of the University of Puerto Rico Medical Sciences Campus on March 14, 2024.

## Case presentation

This is the case of a 42-year-old woman with DM who was hospitalized due to persistent proximal muscle weakness and dysphagia. Two months prior to admission, she was diagnosed with DM at an outpatient clinic, presenting with bilateral proximal muscle weakness in both upper and lower extremities, heliotrope rash, and Gottron’s sign. Initial treatment included prednisone 20 mg daily, azathioprine 100 mg daily, and hydroxychloroquine 400 mg daily. However, these medications were discontinued within a week due to gastrointestinal intolerance. She had no fever, anorexia, weight loss, Raynaud’s phenomenon, cough, dyspnea, or chest pain. Her past medical history was notable for obesity and hypertension managed with irbesartan. There was no family history of autoimmune diseases.

**Figure 1:**
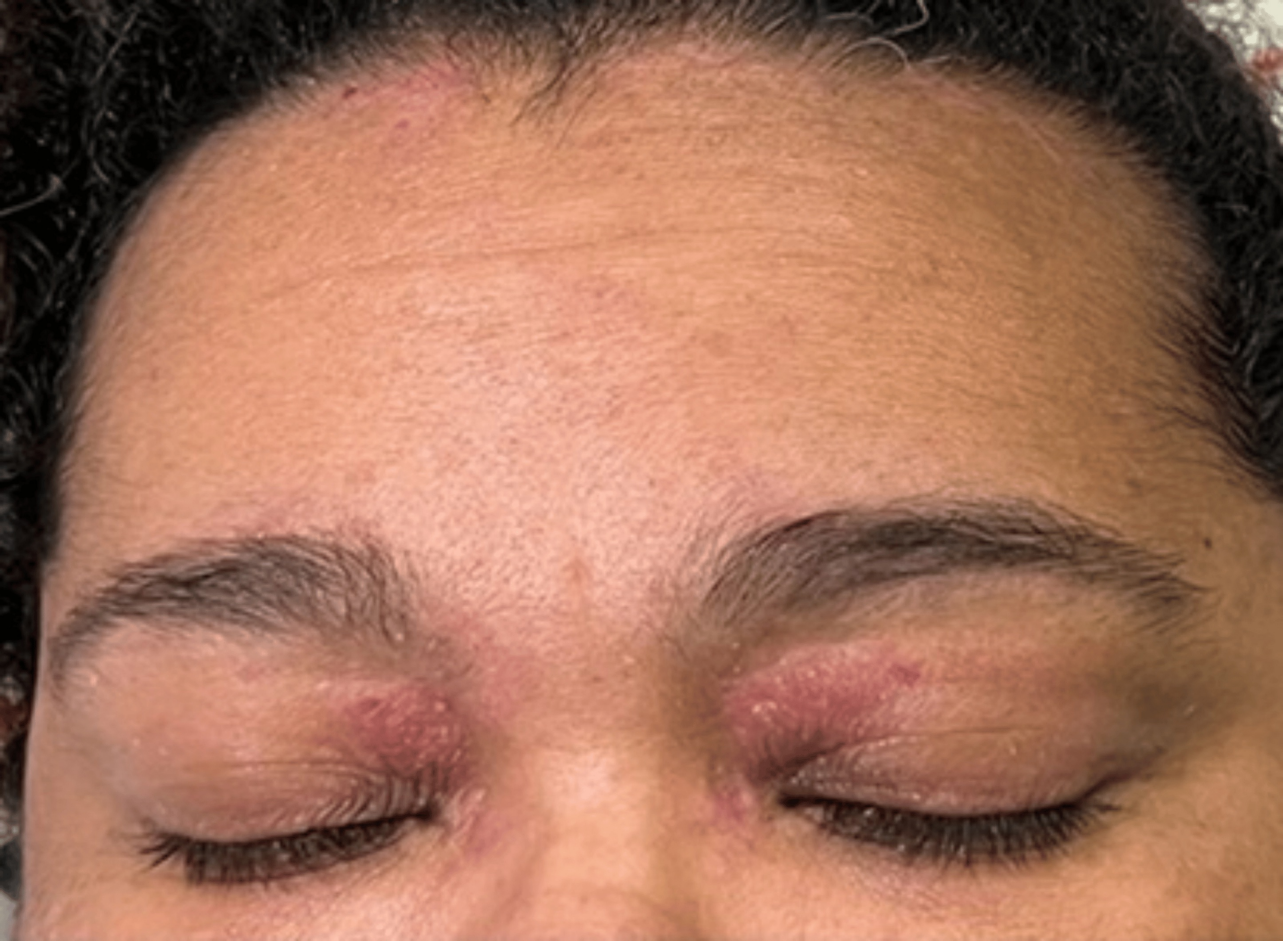
Violaceous discoloration and swelling of the bilateral upper eyelids, consistent with a heliotrope rash

On admission, her vital signs were within normal limits: temperature 36.0°C, blood pressure 124/70 mmHg, heart rate 81 bpm, respiratory rate 20 breaths per minute, and oxygen saturation 98% on room air. Her body mass index was 53.9 kg/m². Skin examination revealed a violaceous rash with swelling of the bilateral upper eyelids consistent with a heliotrope rash (Figure 1), as well as an erythematous macular rash on the neck, upper chest, and arms. She also had a flat, erythematous rash over the bilateral metacarpophalangeal joints (Gottron’s sign) and periungual erythema. Muscle strength was graded 4/5 in the proximal muscle groups of both upper and lower extremities, with relatively greater weakness in the lower extremities. No lymphadenopathy was noted. Pulmonary, cardiovascular, abdominal, and neurological examinations were unremarkable.

**Figure 2:**
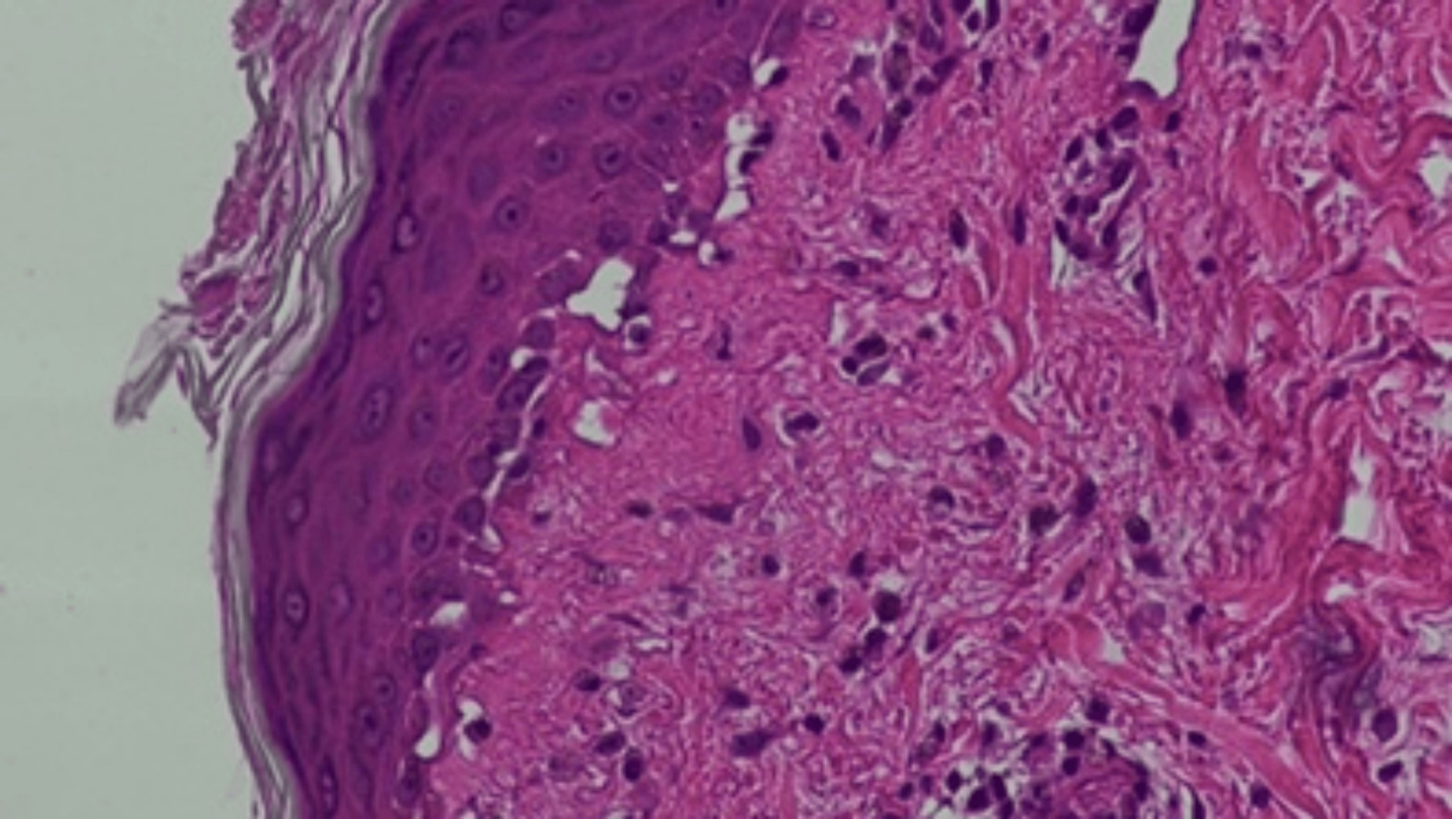
Punch biopsy of an erythematous rash on the upper chest reveals interface dermatitis with focal vacuolar changes and mild dermal mucin deposition, findings consistent with dermatomyositis (Hematoxylin and eosin stain; ×200).

Laboratory studies performed within 48 hours of admission were notable for an elevated serum aldolase level of 24.8 U/L (reference range: 1.0-7.5 U/L) and a creatine phosphokinase (CPK) level of 1063 U/L (reference range: 30-145 U/L). Liver enzymes were markedly elevated, with aspartate aminotransferase (AST) at 394 IU/L and alanine aminotransferase (ALT) at 393 IU/L (reference range: 0-40 IU/L), along with increased gamma-glutamyl transferase (GGT) at 216 U/L (reference range: 3-40 U/L) and lactate dehydrogenase (LDH) at 417 U/L (reference range: 140-280 U/L). Complete blood count, serum creatinine, and urinalysis were within normal limits. An autoimmune myositis panel was positive for anti-MDA5 antibody, while antibodies to Jo-1, PL-7, PL-12, EJ, OJ, SRP, Mi-2 alpha, Mi-2 beta, TIF-1 gamma, and NXP-2 were all negative. A biopsy of the erythematous rash on the upper chest revealed interface dermatitis with focal vacuolar changes and mild dermal mucin deposition (hematoxylin and eosin stain, ×200) (Figure 2).

Alcian blue stain confirmed increased dermal mucin. These findings were consistent with dermatomyositis. Chest radiography showed no acute cardiopulmonary abnormalities. Liver ultrasound revealed mild hepatic steatosis. Computed tomography (CT) of the chest, abdomen, and pelvis performed within the first week of admission showed no evidence of malignancy. Abdominopelvic CT revealed no evidence of organomegaly, masses, or lymphadenopathy. Furthermore, chest CT did not show changes suggestive of ILD such as ground-glass opacities, reticulation, honeycombing, traction bronchiectasis, lung parenchyma nodules, or fibrotic bands. CT angiography of the head demonstrated no vascular abnormalities.

Treatment was initiated with intravenous (IV) methylprednisolone 60 mg every 12 hours for six days, followed by a taper to 70 mg IV daily for three days, and subsequently transitioned to oral prednisone 60 mg daily. Mycophenolate mofetil (MMF) was started at 500 mg orally once daily and later increased to 500 mg twice daily. Due to gastrointestinal intolerance, MMF was discontinued and replaced with mycophenolic acid 720 mg orally twice daily. Dysphagia and proximal muscle weakness improved with treatment, and two weeks later, she was discharged.

**Figure 3:**
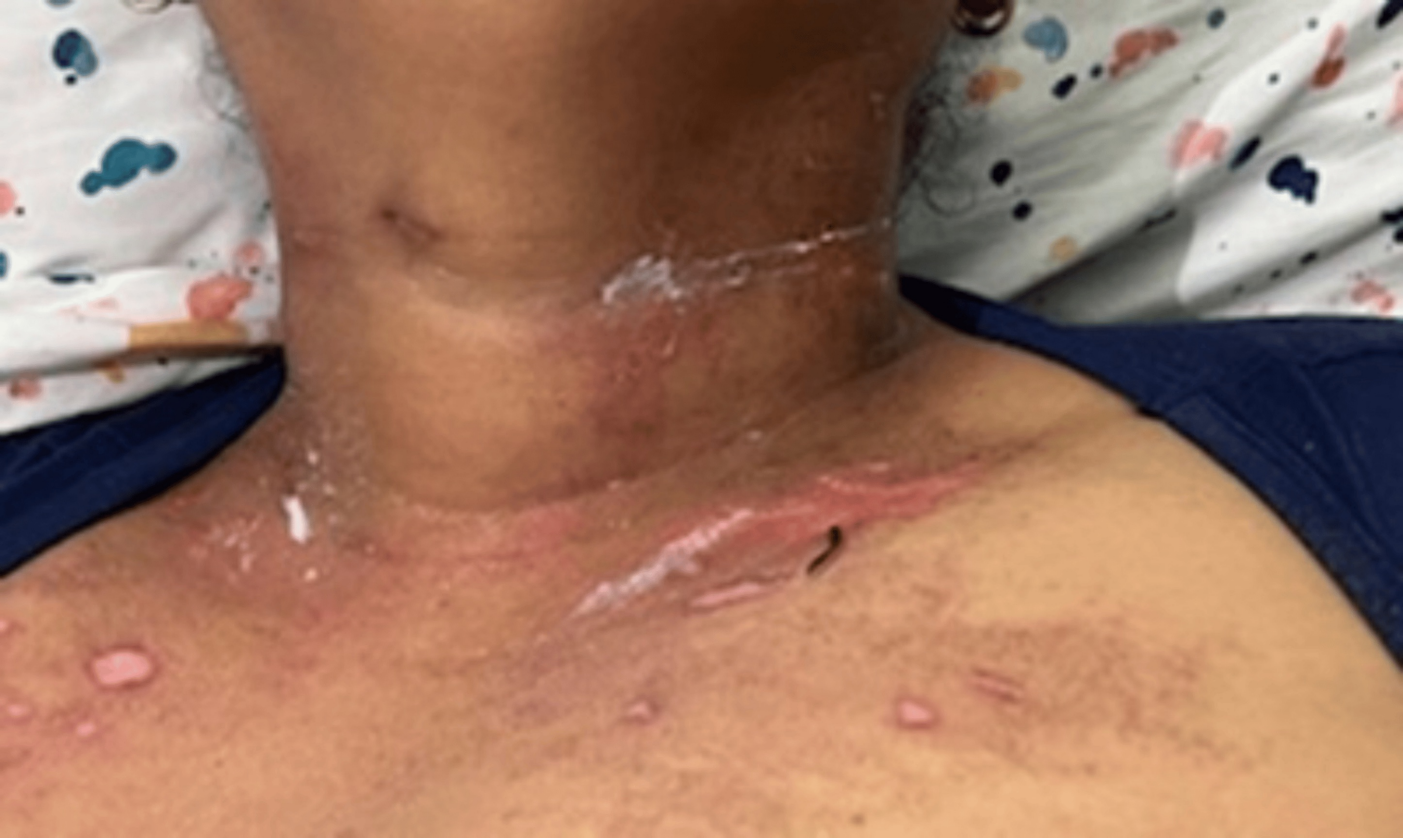
Annular, well-demarcated erythematous plaques observed on the upper chest, consistent with discoid lupus

**Figure 4:**
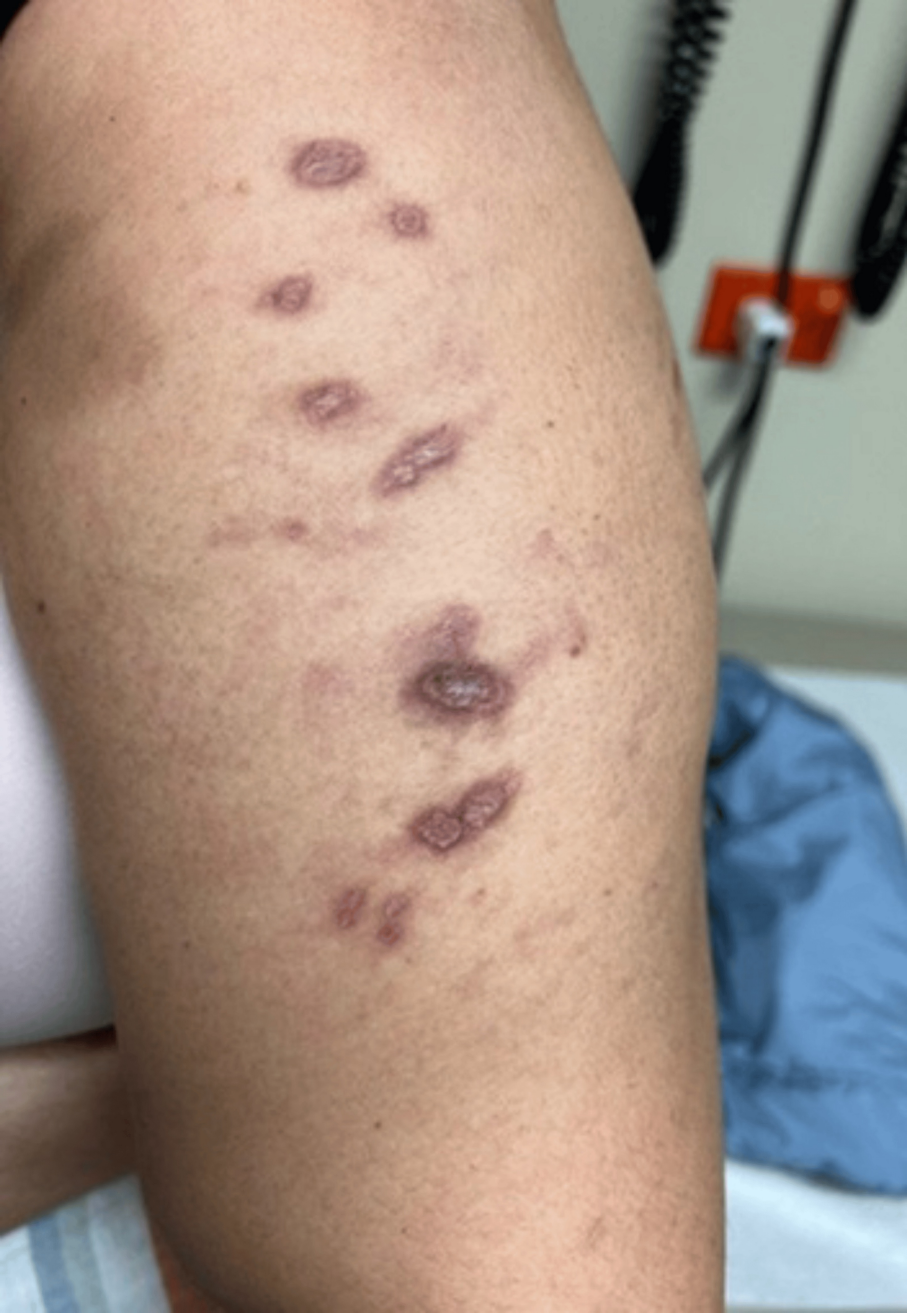
Red-brown, well-demarcated plaques observed on the left upper extremity, consistent with discoid lupus

Two weeks after hospital discharge, the patient was readmitted due to persistent proximal muscle weakness and dermatomyositis-associated skin manifestations, worsening dysphagia, and new-onset symptoms, including oral ulcers, generalized myalgias, polyarthralgia involving the hands, shoulders, and hips, and an annular rash on the chest and upper extremities. On physical examination, her vital signs were within normal limits: temperature 36.2°C, blood pressure 123/73 mmHg, heart rate 68 bpm, respiratory rate 18 breaths per minute, and oxygen saturation 98% on room air. Skin examination revealed previously documented findings, including heliotrope rash, Gottron’s rash, and periungual erythema. Additionally, a new rash was observed on the chest (Figure 3) and upper extremities (Figure 4), characterized by red-brown plaques with well-demarcated borders, consistent with discoid lupus. A single painless ulcer was noted on the inner aspect of the lower lip. Musculoskeletal examination revealed tenderness on palpation of the proximal interphalangeal and metacarpophalangeal joints bilaterally, as well as the wrists and shoulders. Muscle strength remained at 4/5 in the proximal muscle groups of both upper and lower extremities. The remainder of the physical examination was unremarkable.

The laboratory workup at the time of admission is presented in Table 1.

**Table 1:** Summary of laboratory investigations from the second hospital admission AST: Aspartate aminotransferase; ALT: Alanine aminotransferase; GGT: Gamma-glutamyl transferase; LDH: Lactate dehydrogenase; ESR: Erythrocyte sedimentation rate; CRP: C-reactive protein; CCP: Cyclic citrullinated peptide; ANA: Antinuclear antibodies; SSA: Sjogren's Syndrome A; SSB: Sjogren's Syndrome B; RNP: Ribonucleoprotein; ANCA: Antineutrophil cytoplasmic antibodies

Test	Result	Reference range
White blood cell count	3,620/μL	4,500–11,000/μL
Hemoglobin	10.5 g/dL	12–15 g/dL
Platelet count	130,000/μL	150,000–450,000/μL
Lymphocyte count	260/μL	1,500–4,800/μL
Serum creatinine	0.75 mg/dL	0.6–1.2 mg/dL
AST	83 IU/L	0–40 IU/L
ALT	150 IU/L	0–40 IU/L
GGT	149 U/L	3–40 U/L
LDH	313 U/L	140-280 U/L
CPK	310 U/L	30–145 U/L
ESR	44 mm/hr	0–20 mm/hr
CRP	35.5 mg/L	<5 mg/L
Rheumatoid factor	83 IU/mL	0–20 IU/mL
Anti-CCP antibodies	Negative	Negative
C3 complement	82 mg/dL	90–180 mg/dL
C4 complement	22 mg/dL	10–40 mg/dL
ANA	1:2560 (Ro pattern)	<1:40
Anti-Ro/SSA antibodies	>8 U/mL	<1.0 U/mL
Anti-La/SSB antibodies	2.9 U/mL	<1.0 U/mL
Anti-dsDNA antibodies	Negative	Negative
Anti-Smith antibodies	Negative	Negative
Anti-RNP antibodies	Negative	Negative
Anti-topoisomerase I antibodies	Negative	Negative
Anti-smooth muscle antibody	Negative	Negative
Antimitochondrial antibodies	Negative	Negative
ANCA	Negative	Negative
Anti-cardiolipin (IgA, IgG, IgM)	Negative	Negative
Anti-β2-glycoprotein I (IgA, IgG, IgM)	Negative	Negative
Lupus anticoagulant test	Negative	Negative

Urinalysis showed no evidence of proteinuria, hematuria, pyuria, or urinary casts. An esophagogastroduodenoscopy revealed reactive gastritis. Colonoscopy, mammography, and pelvic examination, including cervical cytology, were unremarkable.

Treatment was initiated with IV methylprednisolone 60 mg daily, and mycophenolic acid was continued at 720 mg orally twice daily. Due to worsening dysphagia, she received IV immunoglobulins at a dose of 1 g/kg/day for two consecutive days. Induction therapy with rituximab was also administered at 1,000 mg IV on days 0 and 14, followed by a maintenance regimen of 500 mg IV every six months. Additionally, tacrolimus was added to the treatment regimen at a dose of 5 mg orally once daily.

**Figure 5:**
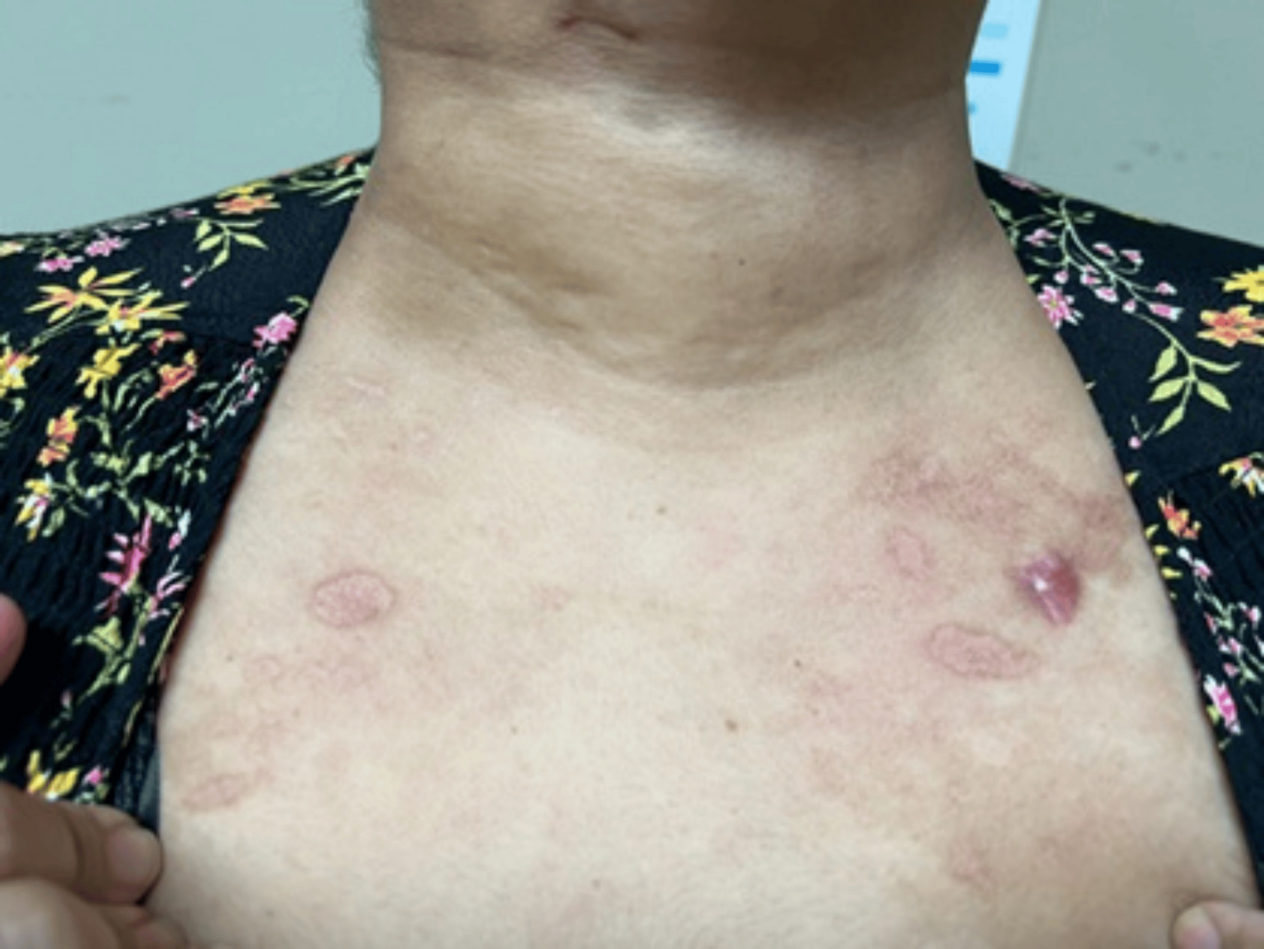
Residual discoid lesions on the upper chest

**Figure 6:**
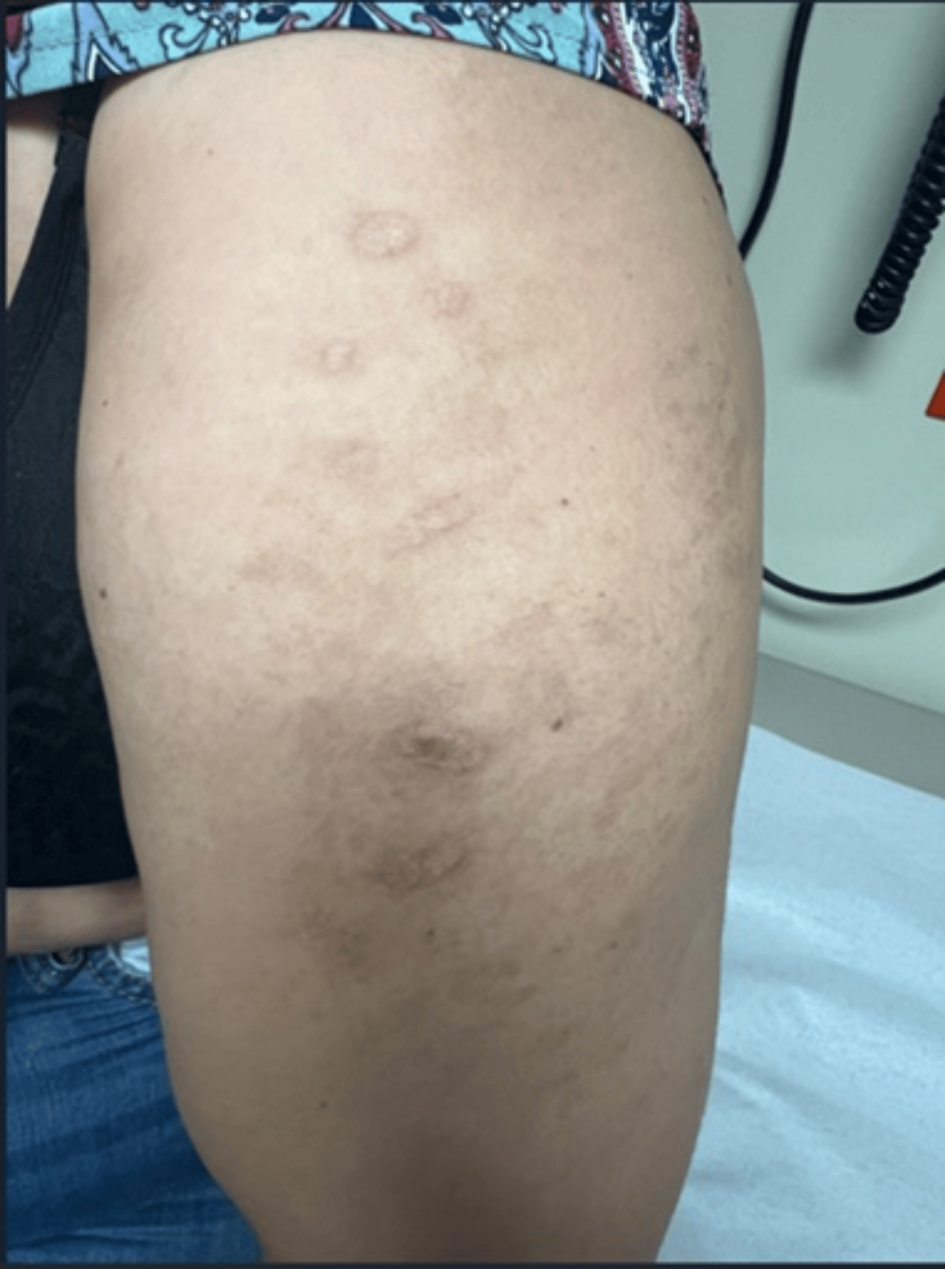
Residual discoid lesions on the left upper extremity

Four weeks after initiation of rituximab induction therapy, the patient experienced resolution of the heliotrope rash, oral ulcer, periungual erythema, polyarthralgia, and dysphagia, along with significant improvement in proximal muscle weakness and generalized myalgias. Mild improvement was noted in Gottron’s sign; however, discoid lesions persisted. At the four-month follow-up, there was marked improvement in the discoid lesions on the chest (Figure 5) and upper extremities (Figure 6), and pancytopenia had resolved. Inflammatory markers, including ESR and CRP, were within normal limits, as were complement C3 levels, CPK, aldolase, AST, and ALT. Oral prednisone was gradually tapered, while mycophenolic acid and tacrolimus dosages remained unchanged. At the 10-month follow-up, prednisone was successfully discontinued. By the 12-month visit, tacrolimus was discontinued, and mycophenolic acid was reduced to 360 mg every other day due to gastrointestinal side effects, including nausea, vomiting, and non-bloody watery diarrhea. Rituximab infusions at 500 mg IV every six months were continued without adverse effects. At the most recent 18-month follow-up, the patient showed no clinical signs of active DM or SLE.

Written informed consent was obtained from the patient to publish this case, including photographs of the skin manifestations.

## Discussion

We present the case of a 42-year-old woman with anti-MDA5 DM who, three months after the onset of DM, developed SLE. Both conditions responded favorably to a combination of immunosuppressive drugs. Table 2 presents the distinguishing clinical features of DM and SLE observed in this case. The differential diagnosis in this patient includes overlap syndrome, mixed connective tissue disease (MCTD), Sjögren’s syndrome, and paraneoplastic myositis. The term overlap syndrome has been defined in various ways by different authors. In general, it refers to a condition in which a patient exhibits clinical features of two or more distinct autoimmune connective tissue diseases (CTDs). Some authors reserve the term for patients who meet the classification criteria for at least two CTDs, while others use it more broadly to describe individuals with features of multiple CTDs, even if they do not fully meet diagnostic criteria for each. Additionally, some definitions require that the features occur simultaneously, while others allow for them to appear at different times. In the present case, the key point is not whether the patient has overlap syndrome per se, but rather that she developed two distinct rheumatic diseases in a sequential manner, with both conditions meeting established classification criteria. MCTD is a consideration; however, the absence of anti-RNP antibodies, which are strongly associated with MCTD, makes this diagnosis less likely. While this patient tested positive for anti-Ro antibodies, the lack of lacrimal and salivary gland involvement argues against a diagnosis of Sjögren’s syndrome. Paraneoplastic myositis is also unlikely, as an extensive malignancy workup was negative. One might consider lymphoma, particularly since pan-CT scans were performed after the initiation of immunosuppressive therapy, including high-dose corticosteroids, which could potentially mask malignancy-related findings. However, this possibility is diminished by the fact that immunosuppressive therapy was eventually significantly downscaled, including the discontinuation of prednisone, without any recurrence or emergence of new symptoms that may suggest lymphoma.

**Table 2:** Clinical features of DM preceding the onset of SLE three months later DM; Dermatomyositis; SLE: Systemic lupus erythematosus; ANA: Antinuclear antibodies; MDA: Melanoma differentiation-associated gene 5

Clinical manifestations of DM	Clinical manifestations of SLE
Heliotrope rash	Discoid lupus rash
Gottron’s sign	Painless oral ulcer
Erythematous rash on the neck, upper chest, and arms	Inflammatory arthritis of proximal interphalangeal and metacarpophalangeal joints, wrists, and shoulders
Dysphagia	Pancytopenia
Bilateral proximal muscle weakness in upper and lower extremities	Positive ANA
Elevated aldolase and creatinine phosphokinase levels	C3 hypocomplementemia
Positive anti-MDA-5 antibody	
Interface dermatitis with focal vacuolar changes and mild dermal mucin deposition on skin biopsy	

Although a muscle biopsy, the gold standard for diagnosing IIM, was not performed, the patient fully met the 2017 European Alliance of Associations for Rheumatology (EULAR)/American College of Rheumatology (ACR) classification criteria for adult and juvenile IIM [4]. Based on this classification, she was categorized as having definite IIM (dermatomyositis subgroup), meeting the following criteria: age at symptom onset ≥40 years, objective, progressive symmetric weakness of the upper and lower extremities, greater weakness in the lower extremities, heliotrope rash, Gottron’s sign, and dysphagia. In addition, a skin biopsy of ulcerative lesions showed histopathologic findings consistent with dermatomyositis, further supporting the diagnosis.

Our patient also met the 2019 ACR/EULAR classification criteria for SLE, with a positive ANA at a titer of ≥1:80, serving as the required entry criterion [5]. She accrued a total of 16 points based on the following features: discoid, inflammatory arthritis, leukopenia, and C3 hypocomplementemia. According to the criteria, a total score of ≥10 is required for SLE classification. Although DM and SLE share several overlapping clinical manifestations, the features observed in this patient are more characteristic of SLE and are uncommon in DM, thereby supporting the diagnosis of concomitant SLE. Although myositis can occur in SLE, this patient exhibited distinctive features of DM, such as heliotrope rash, Gottron’s sign, and anti-MDA5 antibody positivity, supporting a diagnosis of DM-associated myopathy rather than SLE-related myositis.

Anti-MDA5 antibodies, typically associated with ILD and skin ulceration in patients with IIM, were positive in our patient. Anti-MDA5 DM represents a rare subtype of DM, more frequently observed among women and individuals of Asian descent [6]. Unlike classic DM, anti-MDA5-positive DM is not consistently associated with malignancy [7]. Patients with this subtype often exhibit higher disease activity, as reflected by elevated type I interferon (IFN-I) scores, which correlate with poorer outcomes [8]. In our case, the patient did not present with characteristic skin ulcerations or pulmonary involvement at diagnosis or during 18 months of follow-up. Early initiation of immunosuppressive therapy, including high-dose corticosteroids, mycophenolic acid, rituximab, and tacrolimus, all of which are effective in IIM-associated ILD, may have contributed to preventing this severe complication. Notably, anti-MDA5 antibodies are commonly linked to rapidly progressive ILD in clinically amyopathic DM; however, this was not the case in our patient, who initially presented with muscle inflammation.

The coexistence of IIM and SLE is uncommon but has been previously reported. A study examined the occurrence of inflammatory myositis in a cohort of pediatric and adult patients with SLE between 2010 and 2019 [9]. Among 1,718 individuals with SLE, 6.3% had concurrent inflammatory myositis, with higher prevalence noted among individuals of Black race and those with childhood-onset SLE. Similarly, a study by Maazoun et al. reported that myositis occurs in 3.4% of SLE cases, with variable timing of onset [10]. In six patients, five were diagnosed with SLE and DM simultaneously, while one developed SLE nine months after the diagnosis of DM.

In contrast, the association of SLE with anti-MDA5-positive DM is exceedingly rare and has only been described in isolated case reports. To our knowledge, only one other case of coexisting SLE and anti-MDA5-positive DM has been reported. PubMed, Scopus, and Web of Science databases were used to conduct an extensive literature review to identify cases similar to ours. Milam et al. described a middle-aged man who initially presented with DM, characterized by a heliotrope rash, Gottron’s papules, calcinosis cutis, skin ulcers, proximal muscle weakness, and anti-MDA5 antibodies [11]. His initial treatment included prednisone and methotrexate, followed by azathioprine, mycophenolate mofetil, and dapsone. Although muscle weakness resolved, cutaneous manifestations persisted. One year after the DM diagnosis, the patient developed SLE, presenting with mesangial glomerulonephritis, lupus cerebritis, positive antinuclear and anti-dsDNA antibodies, and C3 hypocomplementemia. Unlike our case, SLE onset in that case occurred after the diagnosis of DM, and ILD was present. Another reported case described the coexistence of subacute cutaneous lupus erythematosus (rather than SLE) and amyopathic anti-MDA5-positive DM, complicated by rapidly progressive ILD [12]. In contrast to that case, our patient exhibited not only cutaneous manifestations but also systemic features consistent with SLE. Additionally, her DM was not amyopathic and was not complicated by ILD.

The coexistence of SLE and IIM is not entirely unexpected, as both conditions share key pathogenic mechanisms. These include common genetic and environmental factors, as well as dysregulation of both innate and adaptive immune responses [13,14]. Among these mechanisms, type I IFN plays a particularly important role in the pathogenesis of both SLE and DM. This cytokine drives the activation of genes encoding pro-inflammatory molecules in target cells and is especially implicated in anti-MDA5-positive DM [15]. In this subtype, type I IFN is believed to exert its pathogenic effects primarily through the B-cell activating factor pathway, the formation of neutrophil extracellular traps, and the release of IFN-associated proteins. Given these shared immunopathogenic features, the coexistence of SLE and DM may be biologically plausible. However, it is possible that the use of immunosuppressive therapies, many of which are effective in both conditions, may suppress or delay the clinical emergence of one disease when the other is already being treated.

In addition to shared pathogenic mechanisms and overlapping clinical manifestations, DM and SLE also have significant similarities in their therapeutic approaches [16,17]. Although the coexistence of autoimmune diseases, such as DM and SLE, presents diagnostic and management challenges, many immunosuppressive agents are effective in both conditions. These include glucocorticoids, antimalarials, methotrexate, azathioprine, mycophenolic acid analogs, calcineurin inhibitors, and rituximab. Our patient showed a remarkable clinical response to a regimen that included mycophenolic acid, rituximab, and tacrolimus, which enabled the successful tapering and discontinuation of glucocorticoids. At 18 months of follow-up, she remains in complete clinical remission from both DM and SLE.

## Conclusions

In summary, we present the case of a middle-aged woman with MDA5-positive DM who developed SLE. Although this overlap is rare, it underscores the complex interplay between these two autoimmune diseases and highlights the diagnostic and therapeutic challenges posed by overlapping syndromes. Despite their distinct classifications, DM and SLE share key immunopathogenic pathways, clinical features, and therapeutic options, which necessitate a comprehensive and individualized approach to diagnosis and management. Furthermore, this patient presented with anti-MDA5 antibodies without developing ILD, which is noteworthy, as ILD is a common complication in patients with anti-MDA5-positive DM. Early recognition, revising the diagnosis, particularly in patients presenting new-onset manifestations, and effective treatment require a targeted strategy to address the full spectrum of disease manifestations and to prevent serious complications.
